# Brain-Derived Neurotrophic Factor during Oral Glucose Tolerance Test Predicts Cardiovascular Outcomes

**DOI:** 10.3390/ijms21145008

**Published:** 2020-07-15

**Authors:** I-Te Lee, Yu-Hsuan Li, Wayne Huey-Herng Sheu

**Affiliations:** 1 Division of Endocrinology and Metabolism, Department of Internal Medicine, Taichung Veterans General Hospital, Taichung 40705, Taiwan; itlee@vghtc.gov.tw (I.-T.L.); brightlight720720@gmail.com (Y.-H.L.); 2 School of Medicine, National Yang-Ming University, Taipei 11221, Taiwan; 3 School of Medicine, Chung Shan Medical University, Taichung 40201, Taiwan; 4 College of Science, Tunghai University, Taichung City 40704, Taiwan; 5 Rong Hsing Research Center For Translational Medicine, College of Life Sciences, National Chung Hsing University, Taichung 40227, Taiwan

**Keywords:** area under the curve, brain-derived neurotrophic factor, composite endpoint, oral glucose tolerance test

## Abstract

We investigated if brain-derived neurotrophic factor (BDNF) accumulation after glucose intake could predict cardiovascular outcomes. We enrolled patients admitted for angiography due to angina. After their conditions stabilized, serum BDNF levels were detected at 0, 30, and 120 min during oral glucose tolerance test (OGTT). Area under the curve (AUC) of BDNF was calculated. The first occurrence of nonfatal myocardial infarction, nonfatal stroke, and all-cause mortality served as the primary composite endpoint. Of 480 enrolled patients, 428 completed the follow-up, and 36 primary endpoint events occurred during a median follow-up of 4.4 years. The area under the receiver operating characteristic curve significantly increased from 0.61 (95% confidence interval (CI): 0.52–0.73) for the Framingham risk score (FRS) alone model to 0.72 (95%CI: 0.63–0.81) for the AUC of BDNF plus FRS model (*p* = 0.016) for predicting the primary endpoint, but not to 0.65 (95%CI: 0.55–0.75) for the fasting BDNF plus FRS model (*p* = 0.160). Grouped by median AUC of BDNF of 38.0 (ng/mL) × h, the low BDNF group had a significantly higher risk of the endpoint than the high BDNF group (hazard ratio = 3.410, 95%CI: 1.520–7.653, *p* = 0.003). In conclusion, AUC of BDNF during OGTT could be superior to fasting BDNF for predicting a low cardiovascular risk.

## 1. Introduction

Cardiovascular disease is the major cause of reduced health and has been the largest contributor to disease burden worldwide [1]. Despite much effort focused on preventing cardiovascular disease, the global number of cardiovascular mortality has been increasing [2]. Since cardiovascular disease is associated with a multifactorial pathogenesis and traditional risk factors cannot fully explain individual risk, the identification of new circulating biomarkers is warranted to predict long-term outcomes [3,4].

Brain-derived neurotrophic factor (BDNF), a member of the neurotrophin family, is known to protect neurons and synaptic plasticity [5,6,7]. Several disorders of the central nervous system are associated with a reduction in the circulating level of BDNF and genetic alteration of BDNF [8]. BDNF modulates startle responses, and the Val66Met polymorphism of BDNF has been reported to be a risk variant of vulnerability to stress-inducible illnesses [9,10]. Post-traumatic stress disorder is a risk factor of cardiovascular disease and a predictive factor of death [11,12,13]. Therefore, BDNF may be a bridge between psychiatric disorders and cardiovascular disease [14,15].

Furthermore, low circulating BDNF levels were associated with inflammation and cardiovascular risk in cross-sectional studies [16,17], and found to predict cardiovascular events and all-cause mortality in longitudinal studies [18,19]. BDNF is also associated with energy homeostasis and reported to reduce blood glucose levels in a mouse model of diabetes [20]. Karczewska-Kupczewska et al. [21] reported that circulating BDNF levels decreased after a high-fat meal in young healthy subjects. A reduction in cerebral BDNF output to peripheral blood was noted in young healthy subjects using the hyperglycemic clamp method [22]. The serum level of vascular cell adhesion molecule-1 has been found to be inversely correlated with the serum level of BDNF after glucose intake [16]. Additionally, we previously reported that low BDNF accumulation during a 2-h oral glucose tolerance test (OGTT) was associated with high central pulse blood pressure after the OGTT in subjects aged between 50 and 80 years [23]. Thus, low BDNF accumulation after glucose intake might be associated with cardiovascular disease. We hypothesized that a lower area under the curve (AUC) of BDNF during an OGTT could predict a higher cardiovascular risk and that the prediction might be better than one made using fasting BDNF.

## 2. Results

**Figure 1:**
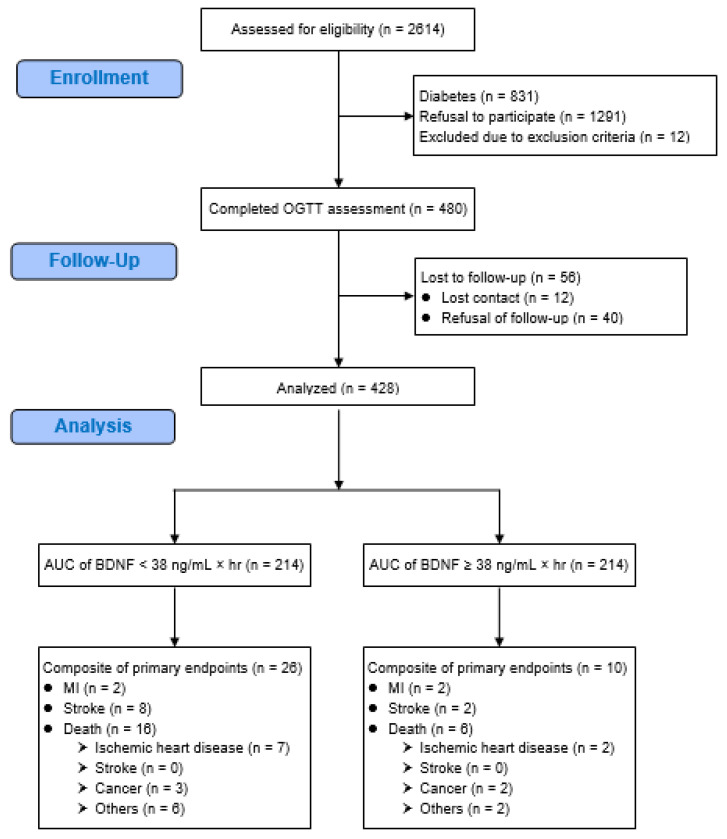
Flow diagram of the enrollment of study patients who had angina. AUC = area under the curve, BDNF = brain-derived neurotrophic factor, MI = myocardial infarction, OGTT = oral glucose tolerance test.

A total of 480 patients were enrolled in the present study, and 428 (89.2%) of them completed the follow-up (Figure 1). Except for a higher proportion of patients using anti-hypertensive drugs (89.5% vs. 73.1%, *p* = 0.001), there was no significant difference in patients who completed the follow-up compared to those who were lost to follow-up (Table 1). During the median follow-up period of 4.4 years, there were 36 primary composite endpoint events, including four myocardial infarction (MI) events, 10 stroke events, and 22 death events.

**Figure 2:**
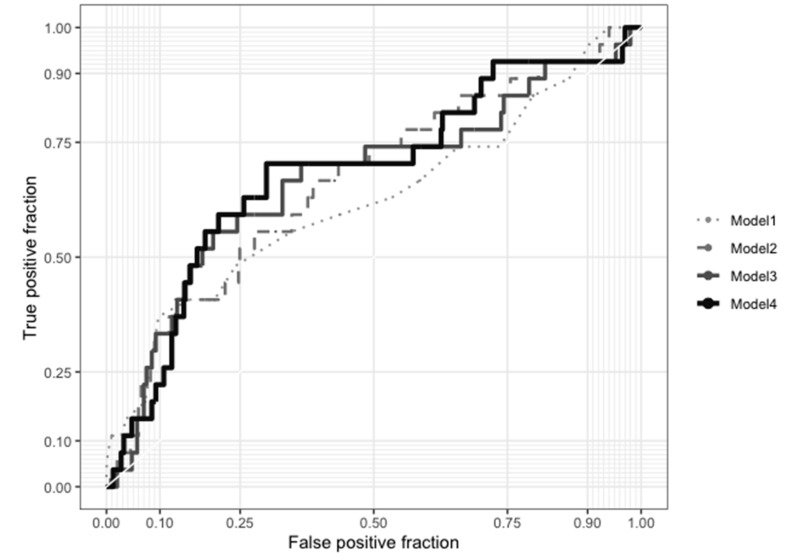
Receiver operating characteristic (ROC) curves for prediction of the primary composite endpoint. Model 1: Framingham risk score (FRS) alone, Model 2: fasting brain-derived neurotrophic factor (BDNF) + FRS, Model 3: BDNF at 30 min + FRS, and Model 4: area under the curve of BDNF + FRS. The area under the ROC curve was 0.61 (95% CI: 0.52–0.73) for Model 1, 0.65 (95% CI: 0.55–0.75) for Model 2, 0.70 (95% CI: 0.61–0.79) for Model 3, and 0.72 (95% CI: 0.63–0.81) for Model 4. The C index showed that compared with Model 1, Models 4 and 3 had significantly greater predictive power (*p* = 0.016 and 0.038, respectively), but not Model 2 (*p* = 0.160).

To assess the effects of adding fasting BDNF, the AUC of BDNF, and the BDNF at 30 min contents to the Framingham risk score (FRS) on the prediction of the primary composite endpoint, we analyzed the increments in the area under the receiver operating characteristic (ROC) curve (Figure 2). The area under the ROC curve significantly increased from 0.61 (95% CI: 0.52–0.73) for the FRS alone model to 0.72 (95% CI: 0.63–0.81) for the AUC of BDNF plus FRS model (*p* = 0.016) and to 0.70 (95% CI: 0.61–0.79) for the BDNF at 30 min plus FRS model (*p* = 0.038). However, the area under the ROC curve was not significantly increased by adding fasting BDNF to the FRS (0.65, 95% CI: 0.55–0.75; *p* = 0.160). Furthermore, the addition of the AUC of BDNF to the FRS yielded a significant integrated discrimination improvement (IDI) (0.039, 95% CI: 0.006–0.123, *p* = 0.007) and a significant continuous net reclassification improvement (NRI) (0.451, 95% CI: 0.062–0.610, *p* = 0.027). The addition of the BDNF at 30 min to FRS also yielded significant IDI (0.039, 95% CI: 0.004–0.131, *p* = 0.013) and NRI (0.393, 95% CI: 0.001–0.548, *p* = 0.040). However, the addition of fasting BDNF to FRS did not yield significant IDI (−0.001, 95% CI: −0.004–0.021, *p* = 0.163) or NRI (−0.027, 95% CI: −0.234–0.158, *p* = 0.751; Table 2).

Table 3 shows the baseline characteristics of patients grouped according to the median AUC of BDNF of 38.0 (ng/mL) × h (range, 10.2–78.7 (ng/mL) × h). There was no significant difference in sex, glucose concentration, or FRS between patients in the low BDNF group and those in the high BDNF group. The proportion of patients who reached the primary endpoint was significantly higher in the low BDNF group than in the high BDNF group (12.1% vs. 4.7%, *p* = 0.009). Patients in the low BDNF group were significantly older than those in the high BDNF group (63 ± 12 vs. 58 ± 11 years, *p* < 0.001). The prevalence of coronary artery disease (CAD) at the baseline (67.8% vs. 57.0%, *p* = 0.028) was significantly higher in the low BDNF group than in the high BDNF group. Fasting plasma insulin (10.6 ± 7.1 vs. 13.7 ± 17.8 µIU/mL, *p* = 0.021) and homeostatic model assessment of insulin resistance (HOMA-IR) index (2.6 ± 1.8 vs. 3.4 ± 5.6, *p* = 0.039) were significantly lower in the low BDNF group than in the high BDNF group. However, the AUC of insulin and the 30-min insulin incremental response at after oral glucose intake did not significantly diff between two groups. The serum levels of total cholesterol (4.3 ± 1.0 vs. 4.5 ± 1.0 mmol/L, *p* = 0.012) and triglycerides (1.4 ± 0.7 vs. 1.7 ± 1.0 mmol/L, *p* < 0.001) were significantly lower in the low BDNF group than in the high BDNF group. The C-reactive protein (CRP) level was significantly higher in the low BDNF group than in the high BDNF group (2.6 ± 2.5 vs. 2.1 ± 2.3 mg/L, *p* = 0.017). The estimated glomerular filtration rate (eGFR) was significantly lower in the low BDNF group than in the high BDNF group (75 ± 22 vs. 79 ± 19 mL/min/1.73 m^2^, *p* = 0.047). Although the proportion of patients using diuretics was higher in the low BDNF group than in the high BDNF group, the proportion of patients using anti-hypertensive drugs did not significantly differ between the two groups (*p* = 0.207).

**Figure 3:**
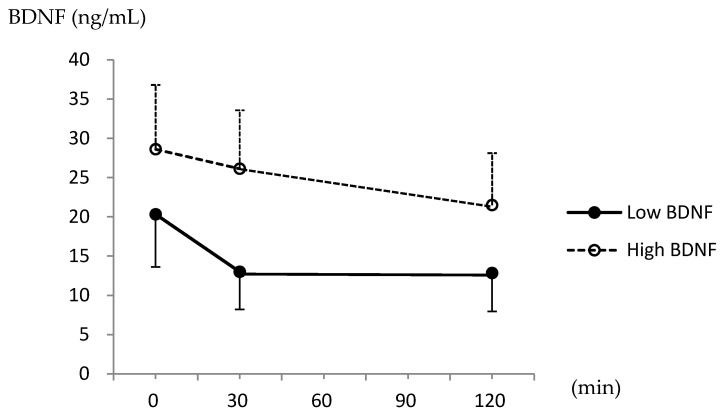
Serum brain-derived neurotrophic factor (BDNF) levels at 0, 30, and 120 min during the oral glucose tolerance test.

Figure 3 shows the changes in serum BDNF levels after the OGTT. Based on the repeated measurements ANOVA, the serum BDNF levels were significantly lower after glucose intake (*p* < 0.001), and there was a significant interaction between BDNF levels and the groups (*p* < 0.001). The rate of the BDNF reduction was significantly greater in the low BDNF group than in the high BDNF group in the first 30 min, and the reduction rate remained significantly different until 120 min, according to the Bonferroni correction for multiple comparisons (−29.3% ± 34.4% vs. −2.8% ± 35.4% at 30 min, and −31.4% ± 31.4% vs. −19.9% ± 31.9% at 120 min; both *p* < 0.001). 

**Figure 4:**
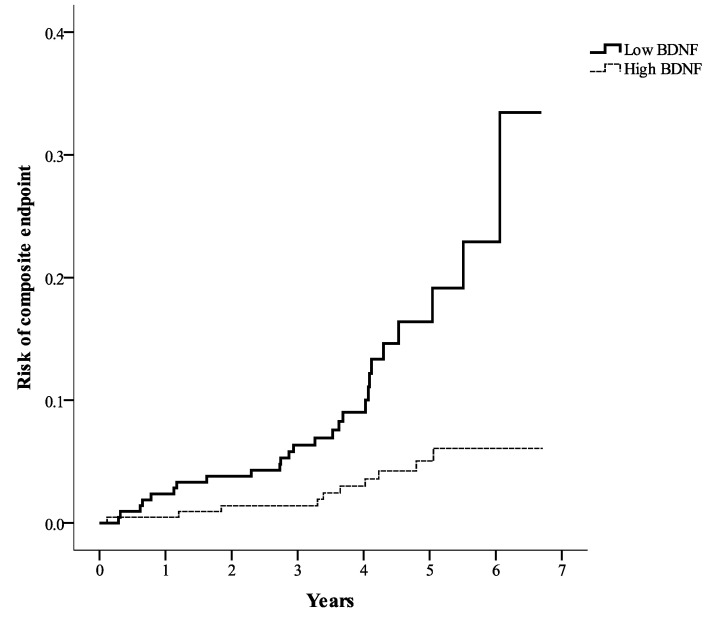
Kaplan–Meier curves showing the composite endpoint of all-cause mortality, nonfatal myocardial infarction, and nonfatal stroke in the high BDNF group and the low BDNF group (log-rank test, *p* < 0.001). BDNF = serum brain-derived neurotrophic factor.

As shown in Figure 4, the risk of the primary composite endpoint was significantly higher in the low BDNF group than in the high BDNF group according to the Kaplan–Meier analysis (log-rank test, *p* < 0.001). To identify the hazard ratio for the primary composite endpoint, multivariate Cox regression analysis was conducted (Table 4). The risk of the primary composite endpoint was significantly higher in the low BDNF group than in the high BDNF group (hazard ratio = 3.410, 95% CI: 1.520–7.653; *p* = 0.003) after adjustments for associated factors selected from Table 3.

## 3. Discussion

Our main finding is that the AUC of BDNF during a 2-h OGTT was predictive of the composite endpoint of nonfatal MI, nonfatal stroke, and all-cause mortality during a median follow-up period of 4.4 years in subjects with angina. To the best of our knowledge, the present study is the first to use dynamic changes in BDNF levels during OGTT to predict cardiovascular outcomes. In line with our results, Jiang et al. [18] reported that a lower morning plasma BDNF level significantly predicted a higher mortality rate in patients with angina, and Kaess et al. [19] also reported that a lower serum BDNF level predicted a greater risk of cardiovascular events in the general population residing in Framingham. A recent genetic study reported that BDNF polymorphisms were associated with ischemic stroke in Chinese individuals with large-artery atherosclerosis [24]. In the present study, the combination of AUC of BDNF and FRS was better than FRS alone for predicting long-term cardiovascular outcomes.

While serum BDNF levels were found to not significantly change after meal intake in children [25,26], a study of adult rats found that brain BDNF expression physiologically increased within 30 min after food intake [27]. We previously demonstrated an obvious change in serum BDNF levels within 30 min after oral glucose intake, and showed that the AUC of the serum BDNF levels at 0, 30, and 120 min was similar to that of the serum BDNF levels at 0, 30, 60, 90, 120 min [28]. We therefore used the former three time points in the present study. Furthermore, we needed to collect blood samples at 30 min for calculating the insulin incremental response to oral glucose, which is a good method to assess insulin secretion [29]. We found that serum BDNF significantly decreased after the intake of 75 g of glucose in adult patients with angina. The percentage reduction in serum BDNF was significantly greater in the low BDNF group, which was associated with a higher risk of the primary endpoint in comparison to the high BDNF group. Esposito et al. [30] reported that 1-h hyperglycemic episodes might increase circulating inflammatory cytokines. Since the inflammation and oxidative stress induced by fluctuating hyperglycemia might be more profound than those induced by persistent hyperglycemia [30,31], it is reasonable to consider that a reduction in serum BDNF would attenuate its protective effect on the endothelium against inflammation induced by the hyperglycemic pulse that occurs during OGTT [32,33].

Although a lower fasting insulin level and a lower HOMA-IR were observed in subjects with a lower AUC of BDNF in the present study, the effects of insulin on the serum BDNF level are not clear. Several studies have reported that the association between the circulating insulin concentration and the BDNF level is not significant [21,22]. We observed that the insulin level after glucose consumption did not significantly differ between the low and high AUC of BDNF groups. BDNF has been reported to improve hepatic insulin resistance in obese rats [34]. However, the association between the AUC of BDNF and cardiovascular endpoints was independent of HOMA-IR in the present study.

BDNF plays an important role in cardiac development and myocardial perfusion [35,36,37], and its deficiency might result in defects of cardiac structure and vasculature [35,36]. Angiogenesis and revascularization could be induced by BDNF in vitro as well as in the mouse model [38,39]. The cardiac infarct area to area at risk ratio was decreased by intra-myocardial BDNF injection in mice subjected to left coronary artery ligation [40]. Therefore, BDNF might prevent myocardium from apoptosis and preserve cardiac function in the ischemic heart [30]. Exercise could increase the circulating BDNF level [41,42], which was also reported to be associated with cardiac angiogenesis and improvement of ejection fraction after MI [43,44].

Arterial stiffness, decreased elastic capacity and cushioning function of the arterial wall, is also an important risk factor for cardiovascular disease. A postprandial vascular dilatation can increase blood flow after meals [45]. Tropomyosin-related kinase receptor B (TrKB), the receptor for BDNF, is expressed in not only the endothelium but also the vascular smooth muscles [35,46]. A low serum BDNF concentration has been reported to be associated with arterial stiffness [47]. We previously reported that a lower AUC of BDNF was associated with higher central pulse pressure after oral glucose intake and that poor postprandial vascular performance might be associated with cardiovascular disease [23].

In the present study, the composite endpoint prevalence was 8.4%, and death was the major event among all the composite endpoints during the median 4.4 years of follow-up. The addition of the AUC of BDNF, but not fasting BDNF, to the FRS significantly improved the prediction of all-cause mortality based on the C index (data was not shown). Interestingly, initial stroke events were more frequent than initial MI in the patients with angina in the present study. However, the rate of MI occurrence is approximately two-fold that of stroke occurrence in patients with acute coronary syndrome based on data from the Taiwan Acute Coronary Syndrome Full Spectrum Registry [48], and data from the Indian subgroup of Long-term Follow-up of Antithrombotic Management Patterns in Acute Coronary Syndrome Patients in Asia (EPICOR-Asia) study [49]. This difference might be due to seven deaths caused by ischemic heart disease in the low BDNF group, two deaths caused by ischemic heart disease in the high BDNF group, and no deaths caused by stroke among the 22 events of all-cause mortality in the present therapy. Furthermore, the majority of patients were followed up in the cardiovascular outpatient center, and aggressive attention to coronary heart disease might have decreased the MI events in the present study.

There are limitations to the present study. First, we only collected blood samples at 0, 30, and 120 min during the OGTT. The serum BDNF levels were still significantly lower at 120 min after glucose intake than at fasting. It has been reported that the reduction in circulating BDNF could persist for at least 6 h after the consumption of a high-fat meal [21]. Second, we observed the protective effect of BDNF on cardiovascular risk during longitudinal follow-up. We did not directly assess the mechanism linking BDNF to cardiovascular protection, which might involve a reduction in inflammation and arterial stiffness based on the results of cross-sectional studies [17,23,47]. Third, we excluded patients with alcohol addiction, but did not quantify alcohol consumption in the enrolled patients. Alcohol abstinence has been reported to increase circulating BDNF levels in patients with alcohol dependence [50,51]. Fourth, we assessed only mature BDNF. The cardiovascular benefits between mature BDNF and precursor BDNF may be different. It has been reported that mature BDNF, but not precursor BDNF, was associated with endothelial function [52]. Fifth, we did not undertake subgroup analyses involving age stratification, which is a confounder associated with the serum BDNF concentration and cardiovascular outcomes. Finally, only Han Chinese patients were enrolled in this study and our findings might not apply to all populations, because ethnic differences in serum BDNF levels have been reported [53].

## 4. Materials and Methods

### 4.1. Study Participants

In this prospective, observational study, we screened adult patients admitted for coronary angiography due to angina between April 2011 and March 2015 in Taichung Veterans General Hospital. We excluded patients with any of the following conditions: (a) a history of diabetes before hospitalization, (b) fasting plasma glucose ≥ 7 mmol/L (126 mg/dL) during hospitalization, (c) acute or chronic infectious diseases, (d) severe systemic disease, such as malignancies, autoimmune diseases and psychiatric disorders, (e) symptomatic congestive heart failure ≥ class 3 as defined by the New York Heart Association [54], (f) addiction to alcohol or drugs, or (g) pregnancy. In a stable condition before discharge, patients were scheduled for a follow-up outpatient visit in an overnight fasting status. A 75gm OGTT for 2 h was performed during the outpatient visit. The study complied with the Declaration of Helsinki and was approved by the Institutional Review Board of Taichung Veterans General Hospital (ethical approval code: C08215B, approval date: 3 February 2009). Written consent was obtained from each patient before the study procedures were performed.

### 4.2. Procedures

Body height and body weight were measured after the participants removed their shoes and any heavy clothing. Blood pressure was detected using the Carescape V100 DINAMAP^®^ Vital Signs Monitor (GE Healthcare, Milwaukee, WI, USA) after the subjects had rested in a seated position for 10 min. After the anthropometric assessments, blood samples were obtained at 0, 30, and 120 min during the OGTT. Fasting blood samples were used to measure glucose, insulin, BDNF, creatinine, CRP, hemoglobin A1c (HbA1c), and lipid profiles; the blood samples collected at 30 and 120 min were used to measure glucose, insulin, and BDNF. AUCs of glucose, insulin, and BDNF were calculated according to their levels at 0, 30, and 120 min.

After the baseline assessments, we followed up the patients by using their electronic medical records in our hospital to collect information on the first episode of all-cause mortality, nonfatal MI, or nonfatal stroke. For patients without any endpoint recorded before December 31, 2017, we arranged a telephone call interview between January 01, 2018 and March 31, 2018. Information on nonfatal MI, nonfatal stroke, or all-cause mortality was collected from the patients themselves or their immediate family members.

### 4.3. Laboratory Assessments

Plasma glucose levels were determined using the oxidase-peroxidase method (Wako Diagnostics, Tokyo, Japan). Plasma insulin levels were determined by using an electrochemiluminescence immunoassay (Roche Diagnostics, Indianapolis, IN, USA). HbA1c levels were determined using boronate affinity high-performance liquid chromatography (NGSP certified; Primus Corp., Kansas City, MO, USA). Serum CRP levels were determined using an ELISA kit (R&D Systems, Minneapolis, MN, USA). Serum creatinine and lipid levels were determined using commercial kits (Beckman Coulter, Fullerton, CA, USA). Human mature BDNF was measured using an immunoassay kit (DBD00; R&D Systems, Minneapolis, MN, USA). The precision of BDNF measurement in serum samples was reflected by an intra-assay coefficient of variation (CV) of 6.2% and an inter-assay CV of 8.1%. The HOMA-IR index was calculated as follows: fasting insulin (µIU/mL) × fasting glucose (mmol/L)/22.5 [55]. The 30-min insulin incremental response to oral glucose was calculated as follows: (insulin_30min_−insulin_0min_ (µIU/mL)) / (glucose_30min_−glucose_0min_ (mmol/L)) [29]. The eGFR was calculated according to the Modification of Diet in Renal Disease equation as follows: 186 × (serum creatinine (mg/dL))^−1.154^ × (age (years))^−0.203^ (× 0.742, if female) mL/min/1.73 m^2^ [56]. Baseline CAD was defined as the presence of one or more of the following: (a) a history of MI, (b) a history of coronary revascularization, and (c) a coronary lesion with lumen narrowing ≥ 50% in angiography. Hypertension was defined as the presence of one or both of the following: (a) a history of antihypertensive agent use and (b) systolic blood pressure ≥ 140 mmHg or diastolic blood pressure ≥ 90 mmHg on the day of the follow-up visit. Chronic kidney disease was defined as an eGRF < 60 mL/min/1.73 m^2^ on the day of the follow-up visit [56]. Normal glucose regulation was defined as plasma glucose < 5.6 mmo/L (100 mg/dL) at fasting and < 7.8 mmo/L (140 mg/dL) at 120 min, and HbA1c < 5.7%. Newly diagnosed diabetes was defined as plasma glucose ≥ 7 mmo/L at fasting or ≥ 11.1 mmo/L (200 mg/dL) at 120 min, or HbA1c ≥ 6.5%. Prediabetes was defined as a glucose status between normal glucose regulation and diabetes criteria [57].

### 4.4. Statistical Analysis

Continuous variables are presented as mean ± standard deviation and categorical data are presented as numbers with percentages. The AUC of BDNF was normally distributed according to the Kolmogorov–Smirnov test. To detect significant between-group differences, we examined continuous variables by using Student’s *t*-test and categorical variables by using the χ^2^ test. Repeated measurements analysis of variance (ANOVA) was used to examine significant differences in glucose and BDNF levels during the OGTT, and the group × BDNF interaction was also examined. The first occurrence of nonfatal MI, nonfatal stroke, and all-cause mortality served as the primary composite endpoint. Based on the previous study in which an estimated hazard ratio of 0.6 was reported when comparing high serum BDNF to low serum BDNF on prediction of cardiovascular risk [19], a sample of 420 subjects divided into two groups using a 1:1 ratio was required to detect differences with a two-sided significance level of 5% and a statistical power of 80%.

FRS was calculated to determine the risk of cardiovascular disease as the standard risk factor [58]. The increases in predictive values of the primary endpoint caused by adding the AUC of BDNF, the BDNF at 30 min, and the fasting BDNF to FRS were assessed using the increments in the area under the ROC curve. The C index was used to compare the areas under the ROC curve between different models. IDI and continuous NRI were also assessed.

The univariate cumulative risk for the primary endpoint was assessed using Kaplan–Meier analysis, and statistically significant differences were tested using the log-rank test. Multivariate Cox proportional hazards regression analyses were used to determine the risk of the primary endpoint according to the AUC of BDNF status. A two-sided *p* value < 0.05 was considered statistically significant. Statistical analyses were performed using SPSS v22.0 (IBM, Armonk, NY, USA), except for the C index, IDI, and NRI using R software v3.4.

## 5. Conclusions

A low AUC of BDNF during the OGTT was an independent predictor for the composite endpoint of nonfatal MI, nonfatal stroke, and all-cause mortality in angina patients during a median follow-up of 4.4 years. The predictive ability of the AUC of BDNF during OGTT might be better than that of fasting BDNF. However, further studies with intervention treatments are needed to determine the causal relationship between the reduction in the serum BDNF level after glucose intake and cardiovascular disease.

## Figures and Tables

**Table 1:** The characteristics of patients who completed follow-up and those who were lost to follow-up.

	Lost to Follow-Up (*n* = 52) Mean ± SD	Completed Follow-Up (*n* = 428) Mean ± SD	*p*
Age (years)	60 ± 12	61 ± 12	0.600
Male, *n* (%)	40 (76.9%)	353 (82.5%)	0.429
Current smoker, *n* (%)	9 (17.3%)	52 (12.1%)	0.404
Previous CAD, *n* (%)	26 (50.0%)	267 (62.4%)	0.114
BMI (kg/m^2^)	25.5 ± 3.3	26.1 ± 3.8	0.230
Systolic BP (mmHg)	126 ± 16	127 ± 18	0.763
Diastolic BP (mmHg)	74 ± 11	74 ± 10	0.933
AUC of BDNF (ng/mL) × h	40.6 ± 15.9	38.5 ± 13.2	0.308
AUC of glucose (mmol/L) × h	17.3 ± 3.2	16.7 ± 3.1	0.191
Glucose at fasting (mmol/L)	5.4 ± 0.7	5.3 ± 0.7	0.347
Glucose at 30 min (mmol/L)	9.4 ± 1.7	9.3 ± 1.6	0.610
Glucose at 120 min (mmol/L)	8.8 ± 2.6	8.2 ± 2.7	0.130
Fasting insulin (µIU/mL)	10.2 ± 7.9	12.1 ± 13.7	0.305
HOMA-IR	2.5 ± 2.1	3.0 ± 4.1	0.393
HbA1c (%)	5.9 ± 0.5	5.8 ± 0.6	0.237
Total cholesterol (mmol/L)	4.6 ± 1.0	4.4 ± 1.0	0.169
LDL cholesterol (mmol/L)	2.7 ± 0.9	2.5 ± 0.8	0.064
HDL cholesterol (mmol/L)	1.3 ± 0.3	1.2 ± 0.3	0.065
Triglycerides (mmol/L)	1.3 ± 0.8	1.5 ± 0.9	0.139
C-reactive protein (mg/L)	2.6 ± 2.4	2.4 ± 2.4	0.505
eGFR (mL/min/1.73 m^2^) *	78 ± 22	77 ± 20	0.912
Statins, *n* (%)	24 (46.2%)	246 (57.5%)	0.160
Antiplatelet agent, *n* (%)	47 (90.4%)	409 (95.6%)	0.200
Antihypertensive agent use, *n* (%)	38 (73.1%)	383 (89.5%)	0.001
ACE inhibitor or ARB	21 (40.4%)	248 (57.9%)	0.024
α-blocker	1 (1.9%)	22 (5.1%)	0.495
β-blocker	20 (38.5%)	124 (29.0%)	0.211
Calcium channel blocker	28 (53.8%)	220 (51.4%)	0.852
Diuretics	8 (15.4%)	71 (16.6%)	0.982
Framingham risk score	13 ± 5	14 ± 5	0.308

* eGFR was logarithmically transformed in the analyses due to skewed distributions. ACE = angiotensin-converting enzyme, ARB = angiotensin II receptor blocker, AUC = area under the curve, BDNF = brain-derived neurotrophic factor, BMI = body mass index, BP = blood pressure, CAD = coronary artery disease, eGFR = estimated glomerular filtration rate, HbA1c = hemoglobin A1c, HDL = high-density lipoprotein, HOMA-IR = homeostatic model assessment of insulin resistance, LDL = low-density lipoprotein, SD = standard deviation.

**Table 2:** Total integrated discrimination index (IDI) and net reclassification index (NRI) for adding fasting brain-derived neurotrophic factor (BDNF), BDNF at 30 min, and area under the curve (AUC) of BDNF to Framingham risk score (FRS) to predict the cardiovascular events.

Model	IDI (95% CI)	*p*	NRI (95% CI)	*p*
Model 1	Reference		Reference	
Model 2	−0.001 (−0.004, 0.021)	0.163	−0.027 (−0.234, 0.158)	0.751
Model 3	0.039 (0.004, 0.131)	0.013	0.393 (0.001, 0.548)	0.040
Model 4	0.039 (0.006, 0.123)	0.007	0.451 (0.062, 0.610)	0.027

Model 1 = FRS alone, Model 2 = fasting BDNF + FRS, Model 3 = BDNF at 30 min + FRS, and Model 4 = AUC of BDNF + FRS, CI = confidence interval.

**Table 3:** The characteristics of patients with high AUC of BDNF and low AUC of BDNF at baseline *.

	Low BDNF (*n* = 214) Mean ± SD	High BDNF (*n* = 214) Mean ± SD	*p*
Age (years)	63 ± 12	58 ± 11	<0.001
Male, *n* (%)	175 (81.8%)	178 (83.2%)	0.799
Current smoker, *n* (%)	19 (8.9%)	33 (15.4%)	0.054
Previous CAD, *n* (%)	145 (67.8%)	122 (57.0%)	0.028
BMI (kg/m^2^)	25.5 ± 3.5	26.8 ± 4.0	<0.001
Systolic BP (mmHg)	126 ± 19	128 ± 18	0.164
Diastolic BP (mmHg)	73 ± 10	76 ± 10	0.001
AUC of BDNF (ng/mL) × h	27.7 ± 6.5	49.4 ± 8.5	<0.001
BDNF at fasting (ng/mL)	20.3 ± 6.7	28.6 ± 8.2	<0.001
BDNF at 30 min (ng/mL)	13.0 ± 4.8	26.1 ± 7.4	<0.001
BDNF at 120 min (ng/mL)	12.8 ± 4.9	21.5 ± 6.6	<0.001
AUC of glucose (mmol/L) × h	16.8 ± 3.2	16.6 ± 2.9	0.497
Glucose at fasting (mmol/L)	5.4 ± 0.8	5.3 ± 0.7	0.198
Glucose at 30 min (mmol/L)	9.2 ± 1.6	9.3 ± 1.7	0.339
Glucose at 120 min (mmol/L)	8.4 ± 2.9	8.0 ± 2.4	0.088
AUC of insulin (µIU/mL) × h	144.5 ± 151.4	145.8 ± 90.1	0.915
Insulin at fasting (µIU/mL)	10.6 ± 7.1	13.7 ± 17.8	0.021
Insulin at 30 min (µIU/mL)	78.0 ± 92.1	80.8 ± 64.2	0.716
Insulin at 120 min (µIU/mL)	85.2 ± 95.0	82.2 ± 59.7	0.695
HOMA-IR	2.6 ± 1.8	3.4 ± 5.6	0.039
Insulin incremental response at 30 min (IU/mol)	18.6 ± 27.0	17.6 ± 14.6	0.636
HbA1c (%)	5.8 ± 0.6	5.9 ± 0.6	0.136
Total cholesterol (mmol/L)	4.3 ± 1.0	4.5 ± 1.0	0.012
LDL cholesterol (mmol/L)	2.4 ± 0.8	2.6 ± 0.9	0.130
HDL cholesterol (mmol/L)	1.2 ± 0.3	1.2 ± 0.2	0.084
Triglycerides (mmol/L)	1.4 ± 0.7	1.7 ± 1.0	<0.001
C-reactive protein (mg/L)	2.6 ± 2.5	2.1 ± 2.3	0.017
eGFR (mL/min/1.73 m^2^) ^#^	75 ± 22	79 ± 19	0.014
Chronic kidney disease, *n* (%)	47 (22.0%)	29 (13.6%)	0.032
Glucose regulation status			0.062
Normal, *n* (%)	61 (28.5%)	84 (39.3%)	
Prediabetes, *n* (%)	47 (22.0%)	41 (19.2%)	
Newly diagnosed diabetes, *n* (%)	106 (49.5%)	89 (41.5%)	
Hypertension, *n* (%)	196 (91.6%)	204 (95.3%)	0.171
Antihypertensive agent use, *n* (%)	187 (87.4%)	196 (91.6%)	0.207
ACE inhibitor or ARB	130 (60.7%)	118 (55.1%)	0.281
α-blocker	13 (6.1%)	9 (4.2%)	0.511
β-blocker	60 (28.0%)	64 (29.9%)	0.749
Calcium channel blocker	108 (50.5%)	112 (52.3%)	0.772
Diuretics	44 (20.6%)	27 (12.6%)	0.038
Statins, *n* (%)	128 (59.8%)	118 (55.1%)	0.379
Antiplatelet agent, *n* (%)	204 (95.3%)	205 (95.8%)	0.999
Framingham risk score	14 ± 5	14 ± 5	0.520
Primary endpoint, *n* (%)	26 (12.1%)	10 (4.7%)	0.009
Nonfatal myocardial infarction	2 (0.9%)	2 (0.9%)	0.999
Nonfatal stroke	8 (3.7%)	2 (0.9%)	0.110
Mortality	16 (7.5%)	6 (2.8%)	0.049

* Patients were grouped according to the median AUC of BDNF value of 38.0 (ng/mL) × h at baseline. ^#^ eGFR was logarithmically transformed in the analyses due to skewed distributions. ACE = angiotensin-converting enzyme, ARB = angiotensin II receptor blocker, AUC = area under the curve, BDNF = brain-derived neurotrophic factor, BMI = body mass index, BP = blood pressure, CAD = coronary artery disease, eGFR = estimated glomerular filtration rate, HbA1c = hemoglobin A1c, HDL = high-density lipoprotein, HOMA-IR = homeostatic model assessment of insulin resistance, LDL = low-density lipoprotein, SD = standard deviation.

**Table 4:** Cox proportional hazards regression analyses for the effects of the associated risk factors on the composite endpoint of all-cause mortality, nonfatal myocardial infarction, and nonfatal stroke.

	Crude				Model 1				Model 2			
	HR	95%	CI	*p*	HR	95%	CI	*p*	HR	95%	CI	*p*
AUC of BDNF < 38 (ng/mL) × h	3.733	(1.780,	7.827)	<0.001	3.051	(1.437,	6.480)	0.004	3.410	(1.520,	7.653)	0.003
Age (years)					1.036	(1.007,	1.065)	0.014	1.043	(1.008,	1.078)	0.014
Male					1.583	(0.558,	4.488)	0.387	1.022	(0.338,	3.087)	0.969
Previous CAD									1.795	(0.786,	4.097)	0.165
BMI (kg/m^2^)									1.095	(0.993,	1.207)	0.068
Diastolic BP (mmHg)									1.005	(0.972,	1.038)	0.786
HOMA-IR									1.045	(0.995,	1.098)	0.081
Total cholesterol (mmol/L)									0.863	(0.584,	1.275)	0.459
Triglycerides (mmol/L)									1.232	(0.810,	1.874)	0.329
C-reactive protein (mg/L)									1.105	(0.992,	1.232)	0.070
CKD *									1.115	(0.503,	2.471)	0.788

* Chronic kidney disease (CKD) = estimated glomerular filtration rate < 60 mL/min/1.73 m^2^. AUC = area under the curve, BDNF = brain-derived neurotrophic factor, BMI = body mass index, BP = blood pressure, CAD = coronary artery disease, CI = confidence interval, HOMA IR = homeostatic model assessment of insulin resistance, HR = hazard ratio.

## Abbreviations

ACE	angiotensin-converting enzyme
ARB	angiotensin II receptor antagonist
ANOVA	analysis of variance
AUC	area under the curve
BDNF	brain-derived neurotrophic factor
BMI	body mass index
BP	blood pressure
CAD	coronary artery disease
CI	confidence interval
CRP	C-reactive protein
CV	coefficient of variation
eGFR	estimated glomerular filtration rate
FRS	Framingham risk score
HbA1c	hemoglobin A1c
HDL	high-density lipoprotein
HOMA-IR	homeostatic model assessment of insulin resistance
IDI	integrated discrimination improvement
LDL	low-density lipoprotein
MI	myocardial infarction
NRI	net reclassification improvement
OGTT	oral glucose tolerance test
ROC	receiver operating characteristic
SD	standard deviation
TrKB	tropomyosin-related kinase receptor B
